# Palms (Arecaceae) and Meligethinae (Coleoptera, Nitidulidae): A Long Evolutionary Journey

**DOI:** 10.3390/plants14162487

**Published:** 2025-08-11

**Authors:** Meike Liu, Jinting Che, Simone Sabatelli, Pietro Gardini, Simone Fattorini, Andrzej Lasoń, Josef Jelínek, Paolo Audisio

**Affiliations:** 1 College of Agriculture, Yangtze University, Jingzhou 434025, China; liumk2009@126.com; 2 MARA Key Laboratory of Sustainable Crop Production in the Middle Reaches of the Yangtze River (Co-Construction by Ministry and Province), College of Agriculture, Yangtze University, Jingzhou 434025, China; wodedipan0319@126.com; 3 Department of Biology and Biotechnologies “Charles Darwin”, Sapienza Rome University, 00185 Rome, Italy; pietro.gardini@uniroma1.it; 4 National Biodiversity Future Center (NBFC), Piazza Marina 61, 90133 Palermo, Italy; 5 Department of Life, Health and Environmental Sciences, University of L’Aquila, Via Vetoio, 67100 L’Aquila, Italy; simone.fattorini@univaq.it; 6 Independent Researcher, ul. Wiejska 4B/85, 15-352 Białystok, Poland; haptos@interia.pl; 7 Department of Entomology, National Museum, Cirkusová 1740, 9-Horní Počernice, CZ-193 00 Praha, Czech Republic; jj.nitidula@seznam.cz

**Keywords:** palms, pollen beetles, dicots and monocots, insect–host plant relationships, host shifts, insect–host plants coevolution vs. sequential evolution, pollination ecology

## Abstract

Arecaceae (palms) constitute a highly diversified family of monocots, distributed especially in tropical and subtropical areas, including approximately 2600 species and 180 genera. Palms originated by the end of the Early Cretaceous, with most genus-level cladogenetic events occurring from the Eocene and Oligocene onward. Meligethinae (pollen beetles) are a large subfamily of Nitidulidae (Coleoptera), including just under 700 described species, and some 50 genera. Meligethinae are widespread in the Palearctic, Afrotropical, and Oriental Regions. All meligethine species are associated with flowers or inflorescences of several plant families, both dicots (the great majority) and monocots (around 7%); approximately 80% of known species are thought to be monophagous or strictly oligophagous at the larval stage. The origin of Meligethinae is debated, although combined paleontological, paleogeographical, and molecular evidence suggests placing it somewhere in the Paleotropics around the Eocene–Oligocene boundary, ca. 35–40 Mya. This article reviews the insect–host plant relationships of all known genera and species of Meligethinae associated with Arecaceae, currently including some 40 species and just under ten genera (including a possibly new African one). The role of adults as effective and important pollinators of their host palms (also in terms of provided ecosystem services) has been demonstrated in some common palm species. All Meligethinae living on palms show rather close phylogenetic relationships with one another and with the mainly Eastern Palearctic genus *Meligethes* Stephens, 1830 and related genera (associated with dicots of the families Rosaceae, Brassicaceae, or Cleomaceae). Molecular data suggests that the palm-associated Paleotropical genus *Meligethinus* Grouvelle, 1906 constitutes the sister-group of *Meligethes* and allied genera. Some hypotheses are presented on the evolution of Meligethinae associated with palms and their probably rather recent (early Miocene–Pleistocene) radiation on their host plants. Meligethinae likely radiated on palms long after the diversification of their hosts, and their recent evolution was driven by repeated radiation on pre-existing and diverse palm taxa, rather than ancient host associations and coevolution. Finally, this article also briefly summarized the relationships that other unrelated groups of Nitidulidae have established with palms around the world.

## 1. Introduction

**Figure 1:**
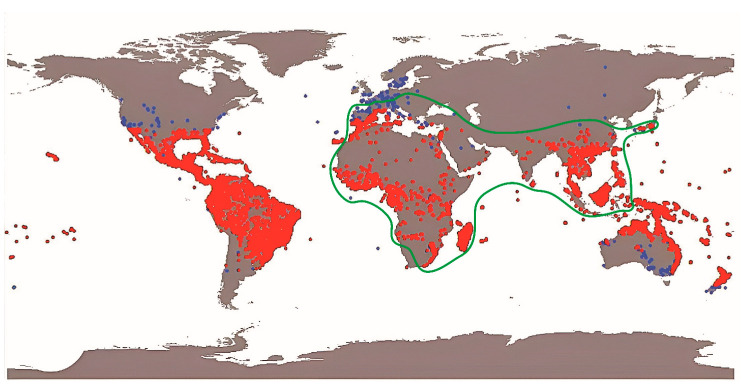
Global distribution of palm species (red points: primary distribution; blue points: secondary distribution, mainly resulting from human-mediated introductions of ornamental or cultivated species) and (green line) known primary and secondary distribution of Meligethinae associated with palms. Redrawn from Reichgelt et al. [8], with permission.

Palms (family Arecaceae) represent a large and highly diversified group of monocotyledonous plants, distributed especially in tropical and subtropical areas, counting worldwide approximately 2600 species and 180 genera (Figure 1) [1,2,3,4,5,6,7]. Members of this clade are widely known for their freeze intolerance (although this varies greatly across different genera and species, organs, and life stages), being able to survive only in areas with coldest month mean temperatures, usually >3 °C, and minimum mean annual temperature of ca. 10 °C [8,9]. Palms likely originated by the end of the Early Cretaceous, around 100 Mya, with most genus-level cladogenetic events occurring from the Middle Eocene (and even more from the late Oligocene) onward [10,11].

**Figure 2:**
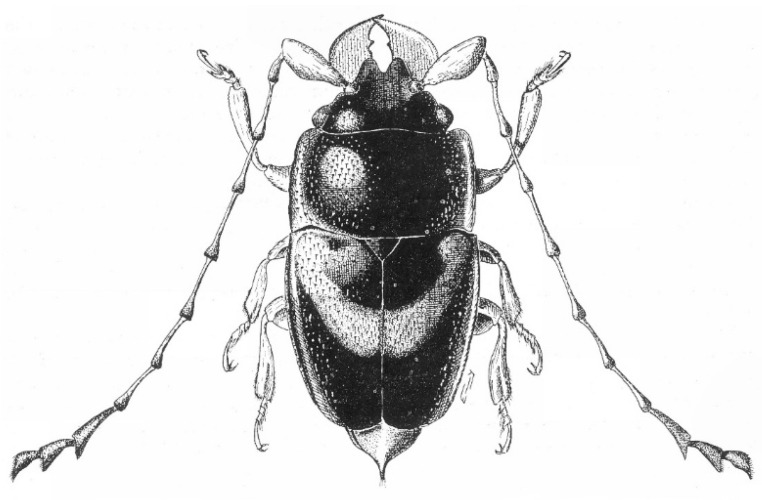
Habitus of a male specimen of *Palmopria elaeidis* S. Endrödy-Younga, 1978, from the Democratic Republic of Congo; body length: ca. 3 mm. From Endrödy-Younga [15].

**Figure 3:**
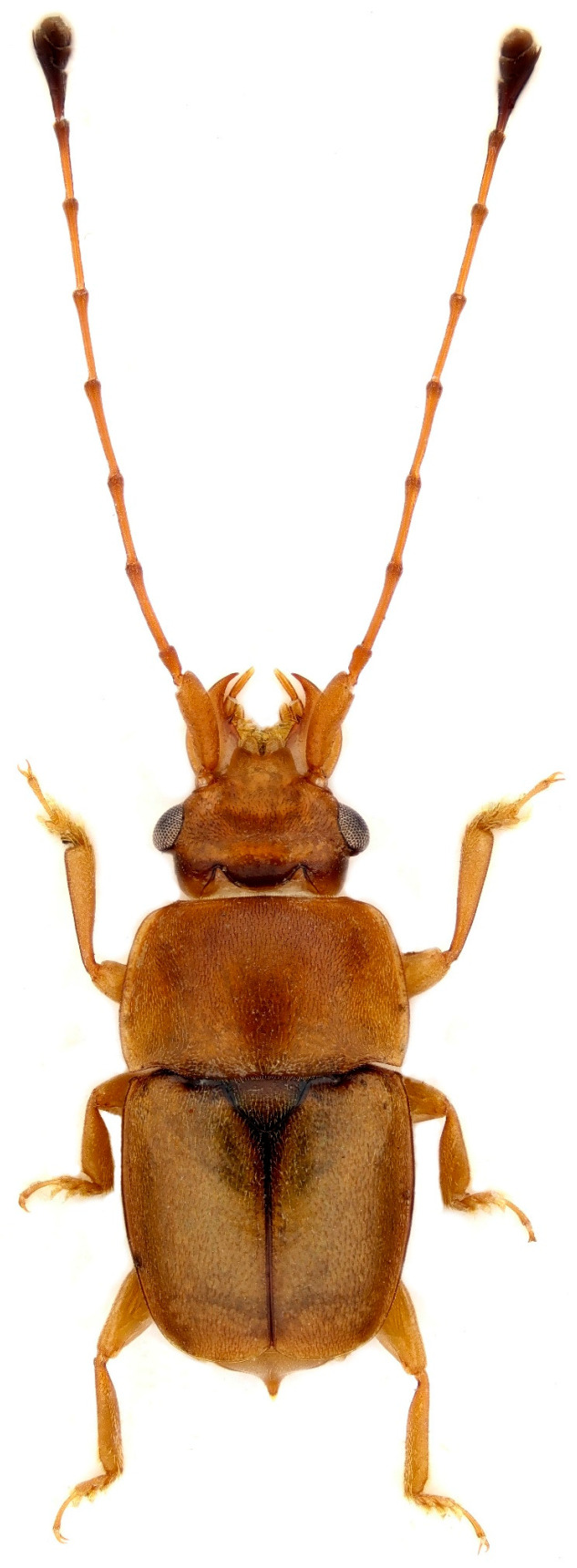
Male specimen of *Microporodes dispar* (Murray, 1864) from Madagascar; body length: ca. 3 mm. Photo by A. Lasoń.

**Figure 4:**
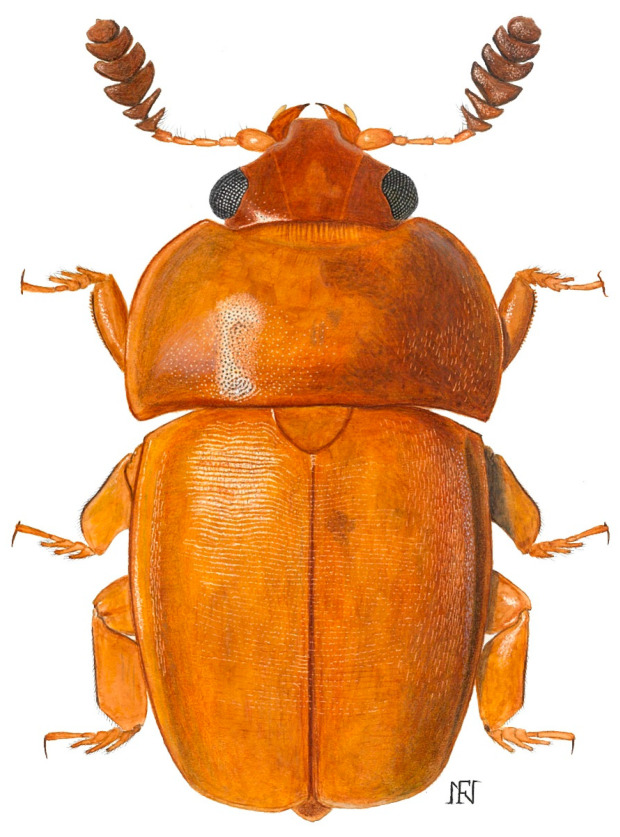
Habitus of a male specimen of *Kabakovia* sp. cfr. *ivoriensis* from Uganda (Audisio et al., unpublished); body length: ca. 3.2 mm. Color plate by Nicoló Falchi.

**Figure 5:**
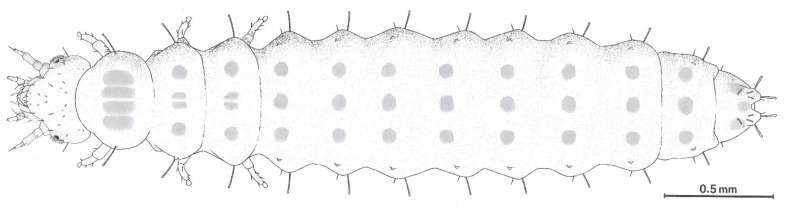
Habitus of a second instar larva of *Meligethinus pallidulus* (Erichson, 1845), reared from male inflorescences of the Western Mediterranean dwarf palm *Chamaerops humilis* L. in Italy. From De Marzo [16].

**Figure 6:**
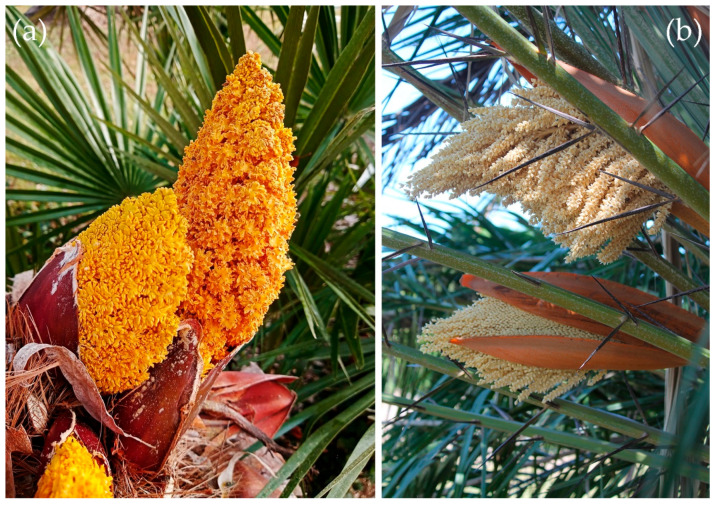
Male inflorescences of palms hosting numerous specimens (larvae and adults) of *Meligethinus* species: (**a**) male inflorescences of the Mediterranean dwarf palm, *Chamaerops humilis* L. from Circeo National Park, Italy, hosting hundreds of individuals (larvae and adults) of *Meligethinus pallidulus* (Erichson, 1845) [17]. Photo by P. Audisio; (**b**) male inflorescences of *Phoenix reclinata* Jacq. from Inhaca Island, southern Mozambique, hosting inside hundreds of individuals (larvae and adults) of five different species of African *Meligethinus* [18]. Photo by S. Sabatelli.

Among the diverse floral visitors and potential pollinators of palms are various beetle clades, including the Meligethinae, a subfamily of Nitidulidae (Coleoptera) commonly referred to as pollen beetles (Figure 2, Figure 3, Figure 4 and Figure 5). Meligethinae include just under 700 described species and are present in almost all the world—except for the Neotropical Region and Antarctica—being particularly numerous in the Palearctic, Afrotropical, and Oriental Regions [12,13,14]. All known meligethine species are closely associated during their larval development with flowers or inflorescences of a wide variety of different plant families, both dicots (the great majority) and monocots (ca. 7% of known species). Despite the ecological and evolutionary interest of this plant–insect association, the relationships between Meligethinae and palms (Figure 6) remain poorly studied and likely underestimated, partly due to limited sampling, the sometimes unpredictable palm flowering phenology, and the meligethine taxonomic complexity.

This review aims to synthesize current knowledge on the ecological and evolutionary relationships between meligethine beetles and palms, with an emphasis on host plant use, pollination roles, biogeographical patterns, and diversification mechanisms. We explore how this plant–insect interaction evolved, whether coevolution played a significant role, and to what extent palms have served as drivers of diversification for the Meligethinae. Finally, we highlight existing knowledge gaps and outline future research directions necessary to better understand this fascinating yet overlooked system.

## 2. Origin of the Meligethinae

The origin of the Meligethinae is still debated, although paleogeographical, paleontological, and molecular clock evidence suggests a Paleotropical origin around the Eocene–Oligocene boundary, approximately 40 Mya [12]. In earlier geological periods, their ecological role of anthophagous beetles was likely occupied by several now-extinct and distantly related clades of other Nitiduloidea lineages, such as ancient genera of Kateretidae, ancient Nitidulidae Epuraeinae, and Nitidulidae Apophisandrinae. The latter may have acted as pollinators for different plant families within the Nymphaeales clade, and possibly even for some Gymospermae and Cycadales [19,20,21].

**Figure 7:**
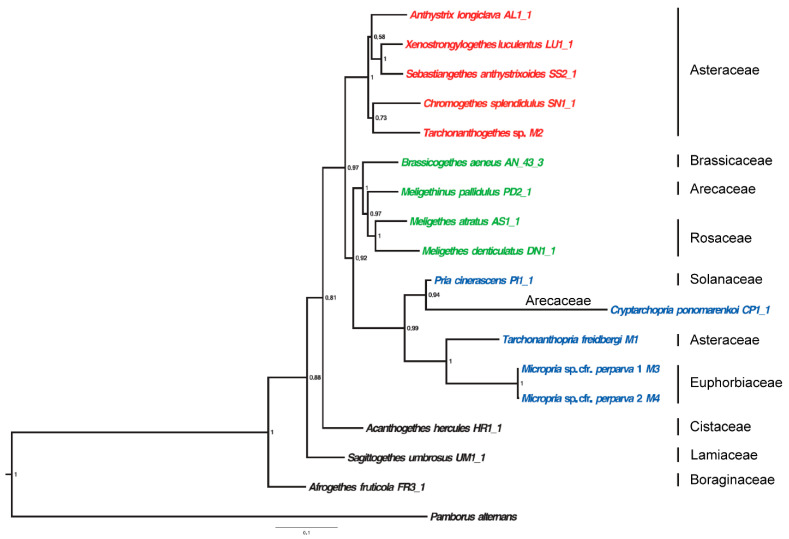
Phylogram (obtained using MrBayes) of selected genera within Meligethinae, representing a significant portion of the lineages relevant to our discussion on the origin of palm-associated Meligethinae. Numbers at the nodes represent Bayesian posterior probabilities. The larval host plant family for each analyzed species is indicated. Each species is assigned to one of the following informal clades of genera: [*Anthystrix* complex of genera + *Chromogethes*] (red); [*Meligethes* complex of genera] (green); [*Pria* complex of genera + *Cryptarchopria*] (blue). Modified and re-drawn, based on original data and methods, from [13].

**Figure 8:**
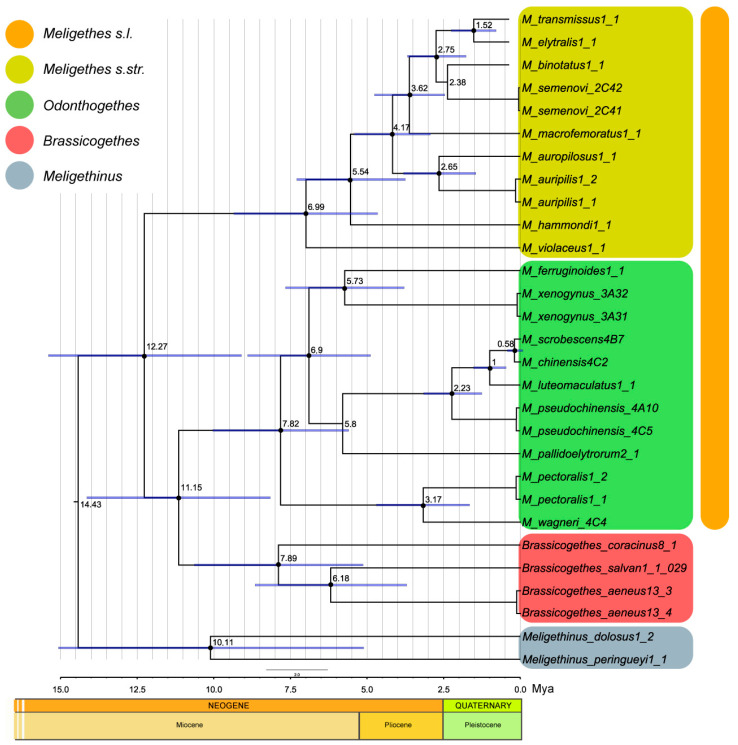
Time-calibrated BEAST phylogeny of representative members of *Meligethes* s.str. *Odonthogethes*, *Brassicogethes*, and *Meligethinus*, inferred from combined mitochondrial sequences (COI, 16S). Numbers at nodes correspond to estimated age (Mya) obtained with calibration of 0.0126 substitutions/site per My; bars represent highest posterior densities (95%) around mean date estimates. Nodes with black dots were supported with high posterior support (>95). From [14].

The earliest fossil genus currently assigned to the Meligethinae is *Melipriopsis* Kirejtshuk, 2011, which includes two related species found in Eocene Baltic amber, dating to approximately 48–34 Mya [22,23,24,25]. However, this genus cannot be confidently placed within the true Meligethinae with certainty, due to several anomalous morphological traits, including the distinctly bordered posterior base of pronotum, the rather acutely sinuated axillary line on the metaventrite (consequently, with a markedly reduced “metasternal axillary space”), and the presence of long cilia along the outer edges of both the pronotum and elytra. All these features are, in fact, inconsistent with the current delimitation of modern Meligethinae [13,14,26]. Similar long cilia are found only in the southern African, sub-eremic and monotypic genus *Sebastiangethes* Audisio & Kirk-Spriggs, 2008, and, even there, they occur only along the outer lateral edges of the elytra. Thus, the oldest probably true fossil meligethine appears to be “*Meligethes*” *detractus* B. Förster, 1891 from the Saxonian Early Oligocene, dated approximately to 30–35 Mya [23,24,25,26,27,28]. A questionable fossil member of the genus *Pria* Stephens, 1830 from Baltic amber (ca 40 Mya), might also represent an early meligethine lineage [29]. All these approximate dates appear to be compatible with available molecular evidence, which estimates the origin of the so-called and clearly quite recent “*Meligethes* complex of genera” at around 15 Mya, while the origin of some of the probably most archaic genera of Meligethinae (such as *Acanthogethes* Reitter, 1871, *Lariopsis* Kirejtshuk, 1989, *Lamiogethes* Audisio & Cline, 2009, and *Afrogethes* Audisio & Cline, 2009) could be dated to around 25–30 Mya [12,13,14,30] (Audisio et al. unpublished data; Figure 7 and Figure 8). Unfortunately, no additional paleogeographical data is available to support a more reliable and accurate estimate of the origin of Meligethinae, which most likely originated somewhere in the Afrotropical Region and subsequently dispersed into Europe and Asia.

On the other hand, there is still an almost complete lack of molecular data to accurately estimate the origin of the subfamily. Based on available evidence, the Meligethinae appear to be the sister-group of the “Nitidulinae 2” group of Lee et al. [31], which corresponds to the so-called “*Soronia* complex of genera” (known as fossil at least from Paleogene), making the Nitidulinae a paraphyletic group [20,31,32]. In fact, the “subfamily” Nitidulinae, as currently delimited, is a heterogeneous and paraphyletic assemblage that will likely require reclassification in the near future into a small number of credibly monophyletic subfamilies [31,33], one of which would obviously be represented by the Meligethinae.

The combined estimates of the origins of palms and the Meligethinae discussed above suggest that the latter emerged during a geological period in which a large part of the Arecaceae had already diversified. This timing would have allowed the Meligethinae to exploit a pre-existing, broad, and diversified ecological and phylogenetic space, especially during the last 20 My, through a model of sequential evolution [34,35,36]. However, this observation does not preclude the possibility of limited and recent instances of true coevolution [37] between the Meligethinae and the Arecaceae, particularly within the last 10–15 My. This mixed evolutionary pattern is consistent with recent molecular evidence from the genera/subgenera *Meligethes* Stephens, 1830, and *Odonthogethes* Reitter, 1871, in relation to the evolutionary history of their Rosaceae host plants (especially of the speciose genera *Rosa* L. and *Rubus* L.) in the Eastern Palearctic [14] (Liu et al. unpublished data). In these cases, as well, the main diversification and evolution of the host plants clearly preceded that of their associated pollen beetles, which likely underwent more recent species-level diversification associated with both *Rosa* and *Rubus* evolution in montane areas of China, Nepal, and surrounding areas, during the last few million years [14].

## 3. The State of the Art of Meligethinae Diversification and Taxonomy

Until a few years ago, the vast majority of known Meligethinae species (>500 out of <700) were attributed to the vast genus *Meligethes*. However, both morphological and molecular evidence revealed that the *Meligethes* represented a heterogeneous and clearly polyphyletic “wastebasket taxon” [12,13,26,38,39]. In response, Audisio et al. [12] tentatively revised and split this taxon into approximately twenty distinct genera, most of which roughly corresponded to the former subgenera or major species-groups previously recognized within the *Meligethes*. Within each of these lineages, most species tend to share the same larval host plant family.

A recent paper [40] proposed a number of new synonymies across various genera within the subfamily Meligethinae, based on very limited data and a simplistic approach. The authors of this article also advocated for the reintroduction of an earlier classification system for the subfamily—one that has since been proven to be untenable, especially in the light of molecular evidence [13,14,41,42]. Given these shortcomings, the taxonomic conclusions presented in that article are not considered further here.

Following the taxonomic and phylogenetic review by Audisio et al. [12]—which will still require additional studies, re-analysis, and refinements using more advanced (e.g., metagenomic) approaches—the nominotypical “subgenus” *Meligethes* (together with the closely related subgenus or genus *Odonthogethes*) is currently understood to include about seventy species from the Palearctic and Oriental regions, all closely associated, during their larval development, with the flowers of Rosaceae [13,14,41,42]. As demonstrated in a series of recent papers [12,13,14,39,43,44,45,46], *Meligethes*, *Odonthogethes*, and *Brassicogethes* Audisio & Cline, 2009, form a well-defined and monophyletic clade (Figure 7 and Figure 8), mainly distributed in the Palearctic region. This clade includes just under 110 species, all of which are associated with particular host plant families, *Meligethes* and *Odonthogethes* with Rosaceae (within the clade Eurosids I, order Rosales) and *Brassicogethes* with Brassicaceae and Cleomaceae (within the clade Eurosids II, order Brassicales).

Sister to this small but speciose clade (then, only including the three genera *Meligethes*, *Odonthogethes*, and *Brassicogethes,* each of them comprising several dozen species) is the palm-associated genus *Meligethinus* Grouvelle, 1906 [12,13,14,43] (Liu et al. unpublished metagenomic data). This genus is also related to a group of other small- to mid-sized meligethine genera known from the Palearctic, Oriental, and Afrotropical–Malagasy regions, including, among others, *Kabakovia* Kirejtshuk, 1979, *Cryptarchopria* Jelínek, 1975, *Horakia* Jelínek, 2000, *Pria* Stephens, 1830, *Tarchonanthopria* Audisio & Cline, 2014, and the tentatively introduced *Microporum* Waterhouse, 1859 group of genera [12,13,14,15,40,43,47,48,49,50,51,52,53,54]. It is likely that an ancient common ancestor of these genera shifted from dicots to a distantly related monocot plant family. This host shift may have triggered a rapid adaptive radiation into a newly available ecological and phylogenetic space (i.e., the highly diverse monocot plants), following evolutionary trajectories similar to those discussed elsewhere [55,56,57,58]. Among these probably more recently originated genera, two small lineages show evident specializations: the *Meligethinus* group of genera on male inflorescences of Arecaceae (palms) (Figure 6a,b) and a few genera of the *Microporum* group on inflorescences of Pandanaceae (screw pines) [12,13,15,17,47,48,49,50,53,54,59]. In particular, several mostly Oriental and Afrotropical species within the *Meligethinus* complex of genera (Table 1) are known to be associated with palms during both larval and adult stages [12,13,40,54,59,60]. However, some recent reviews [6,61] appear to have overlooked the important role of the Meligethinae in palm pollination and conservation.

## 4. The Dicot–Monocot “Host Jump”

As noted elsewhere [67,68,69,70], the number of phytophagous (and/or anthophagous) insect species tends to be positively correlated with the diversity of their host plant taxa. This suggests that when a group of insects undergoes a “host jump” to another new, phylogenetically distant group of plants, they may gain an evolutionary advantage over competitors. If the new host plant group represents an ecologically and trophically open niche—providing abundant, annually stable, and phylogenetically diversified resources—this can facilitate a rapid radiation of the insects within the newly colonized plant lineage [71,72]. Such a model may explain the early evolution of Meligethinae, which first made a “host jump” from dicots to monocots, quickly colonizing an already well-diversified group, the palms. This evolutionary trajectory seems to be recurrent in phytophagous insects, since similar evolutionary phenomena involving recurrent and independent host jumps from dicots to monocots have been documented, e.g., in Chrysomelidae [73]. Recent research indicates that strict cospeciation events are relatively rare (ca. 7%) among phytophagous insects [56]. Instead, cases of “sequential” evolution and adaptive radiation following host shifts to novel, distantly related and already highly diversified host groups are more common [73].

In this context, it has recently been emphasized [67,74,75,76] that similar evolutionary patterns in insect–plant interactions are consistent with the earlier observations of Janzen [75,76] on the theoretical parallel between the evolution of phytophagous insects and principles of insular biogeography. According to this view, when a phytophagous insect clade colonizes a new, phylogenetically distant but highly diversified “plant archipelago”, a rapid adaptive radiation within that host group is likely to occur.

## 5. The Evolution of the Meligethinae on Monocots and Palms

As regards the identity of the ancestral lineages from which the dicot-to-monocot ecological shift—or, more precisely, the “host jump” [56,77]—occurred among the Meligethinae, probably around 20 Mya, the best candidates are almost certainly to be found among the present-day members of the “*Anthystrix* complex of genera”. This group [also] includes the Oriental *Cyclogethes* Kirejtshuk, 1979, and the related Afrotropical genus *Chromogethes* Kirejtshuk, 1989, both associated, in the larval stage, with inflorescences of Asteraceae [12,13,26,38,41,42,78]. Species within the “*Anthystrix* complex of genera” exhibit clear molecular and morphological evidence of phylogenetic relatedness to most of the monocot-associated Meligethinae ([13,39,41,60]; Figure 7), supporting their likely ancestral role in the host shift event. It is also worth noting that a few other meligethine lineages—unrelated to each other—have independently made similar, though more limited, “host jumps” from dicots to monocots. Notable examples include *Restiopria* Audisio, 2011 (a genus comprising a single known species from the southern African Cape Province, associated with prostrate Restionaceae), and *Afrogethes heteropus* (Gerstaecker, 1871), a phylogenetically isolated Afrotropical species found in Central and Western Africa and associated with Poaceae [13,17,79,80].

Based on clear morphological evidence, the genus *Pria* appears not distantly related to all meligethine genera associated with palms (Table 1; Figure 7) or with other monocots (such as the genus *Microporum*, found in the western Indian Ocean islands and associated with the inflorescences of Pandanaceae). *Pria*, which includes around 80 species primarily across the Paleotropics [49], probably shares a far common ancestor with *Meligethinus* and its allied genera, as well as with the above listed members of the “*Anthystrix* complex of genera” (Figure 7) [12,13,49]. Species of *Pria*—a genus distinguished by the absence of the pair of large semicircular impressions on the last abdominal ventrite, a feature present in nearly all other Meligethinae, except *Palmopria* and allied Afrotropical genera, and Oriental *Horakia* + *Cryptarchopria*—have undergone multiple host shifts into dicot lineages. These shifts have led to radiation into several plant families, particularly Solanaceae, Ericaceae, Mesembryanthemaceae, Asteraceae, and possibly also Buddlejaceae [12,13,49], (Audisio et al. unpublished data). *Afrogethes*, *Lamiogethes*, *Lariopsis*, and also some basal members of the “*Anthystrix* complex of genera” were already well differentiated by approximately 25–30 Mya [30]. Possibly around 20 Mya, a lineage phylogenetically related to *Meligethinus* and its relatives likely shifted to monocot hosts, including Arecaceae and Pandanaceae, probably within the Paleotropical region. It is particularly noteworthy that members of the *Meligethes* complex of genera—the recognized sister group of *Meligethinus*—appear to have undergone a retrograde “host-jump” back from monocots to dicots (Rosaceae and Brassicaceae) in the Eastern Palearctic [13,14]. This interpretation is consistent with the combined molecular, morphological, and biogeographical evidence currently available (Figure 7 and Figure 8).

It is also important to note that our knowledge of palms-associated Meligethinae is probably far from complete, due to the rarity of certain palm species and the limited opportunities for entomologists to encounter them in bloom—an essential condition for collecting associated beetles. As a result, new palm-associated taxa continue to be occasionally discovered in tropical areas ([18,40,62]; Table 1). An emblematic example of this knowledge gap concerns the iconic and rare giant palm *Raphia australis* Oberm. & Strey, commonly known as the Kosi palm or *umVuma* (in Zulu). This threatened palm occurs in a very limited area between the southern Mozambique and northeastern South Africa [81,82,83,84,85]. *Raphia australis* is monoecious and monocarpic, producing its massive male and female inflorescences only once in its lifetime. Despite a series of research projects focused on insect biodiversity associated with local palms, our team was unable to locate flowering individuals in southern Mozambique in recent years due to the unpredictability and brevity of their flowering period [18]. Because of the large number of palm species endemic to tropical Africa and to southeastern Asia, from northeastern India to the Philippines and Indonesia, new palm-associated species and genera of Meligethinae are likely to be discovered as more intensive fieldwork and taxonomic studies are undertaken in these areas.

Returning to the key genus *Meligethinus*—which probably represents the starting point for the adaptive radiation of Meligethinae on palms—all members of this predominantly Paleotropical clade (Table 1) appear to be strictly associated with the male inflorescences (spathes) of palms during their larval development. Adults are also rarely found outside these inflorescences, typically only after the usually brief flowering period of the respective host plants has ended [63]. *Meligethinus* has been regarded as perhaps the most archaic of all palm-associated Meligethinae [40,53,54] due to several plesiomorphic traits it shares with other Meligethinae, such as members of the previously mentioned *Meligethes* genus complex. Most species within this clade appear to be strictly monophagous, although a few exhibit oligophagy [12,16,17,18,62,86,87] (Liu & Audisio, unpublished data]. Some species of *Meligethinus* (e.g., *M. pallidulus* Erichson, 1845 from the southwestern Mediterranean, *M. tschungseni* Kirejtshuk, 1987 from China, and some widespread African species) also act as important pollinators of cultivated or ornamental palms, such as *Chamaerops humilis* L., *Trachycarpus fortunei* (Hook.) H. Wendl., *Elaeis guineensis* Jacq. and *Phoenix reclinata* Jacq. (Table 1; Figure 6a,b) [17,18,62,87,88]. Some meligethine genera and species certainly provide notable ecosystem services, particularly in agricultural contexts. In natural ecosystems, most *Meligethinus* species play a major role in the pollination of native palms, including species of conservation concern, from the southwestern Mediterranean (Figure 6a) through the Near East, as well as in the Oriental and Afrotropical regions [16,17,18,54,62,88,89,90,91] (Audisio et al. unpublished data). Therefore, it would be particularly important to determine whether an as yet undiscovered species of *Meligethinus* (or of a related genus) might be involved in the pollination of the aforementioned rare and threatened southern African Kosi palm (*Raphia australis*). Such a discovery could have significant implications for the species’ survival and inform future conservation strategies.

Finally, when examining the relationships between the relatively large genus *Meligethinus* and its palm host plants, it is noteworthy that these interactions are consistent with recent findings from other host–parasite systems, in which coevolution between hosts and parasites is rarely a major driver of speciation [37], except in cases of obligate pollination mutualisms, where close co-adaptation may be involved [57,92,93,94]. In this context, it is interesting to note that some palm species (e.g., *Elaeis guineensis* and *Phoenix reclinata* in tropical Africa: Table 1) [18,62] may simultaneously host larvae and adults of up to five species of *Meligethinus*, even in the same locality and on the same individual palm; these pollen beetle species are often not strictly related phylogenetically, as on the contrary one might expect in the case of sympatric speciation. This evidence suggests that the evolution of Meligethinae on palms has been shaped more by a combination of independent allopatric speciation events, subsequent geographical overlap through range expansion, and repeated host shifts (or “host-switching” [95]) rather than by coevolutionary processes. This hypothesis seems to be strongly strengthened by repeated observations that demonstrate how local multi-specific associations of *Meligethinus* on individuals of the same palm species are highly dynamic, varying from location to location throughout tropical Africa; this circumstance well supports the assumption that (at least) in tropical and equatorial Africa, where the presence of many palm species in the same area is very common, each *Meligethinus* species has a notable propensity to easily colonize new palm hosts, even when the latter are not at all phylogenetically related to one another. In fact, the few apparently strictly monophagous species of this genus are only those that live in areas marginal to the main palm range (Figure 1), such as *Meligethinus pallidulus* in the western Mediterranean coastal maquis, *M. gedrosiacus* in the Iranian–Arabian deserts, and the common *M. tschungseni* in central China forests (Table 1), where only a single native palm species (in the latter case *Trachycarpus fortunei* (Hook.) H. Wendl.) is present. This interpretation is also consistent with the “oscillation hypothesis” of host plant range evolution and speciation in phytophagous insects, which emphasizes dynamic shifts between host specialization and generalization over evolutionary time and space [96,97,98].

## 6. Palms and Other Pollen-Eaters, Pollinators, or Inflorescence-Frequenters Nitidulid Beetles

For the sake of completeness, it is also important to note that in Central and Southern America, where the Meligethinae are absent, palms host a large variety of other anthophagous and pollinating Nitidulidae, mostly represented by the ecologically and geographically vicariant tribe (or subfamily) Mystropini [62,99,100,101,102,103,104,105,106]. When considered as a tribe, the Neotropical Mystropini (which share several markedly convergent external traits with Old World Meligethinae, especially with those analogously developing on palm inflorescences), are currently classified within the subfamily Nitidulinae, a paraphyletic group, as discussed above [31,33].

Finally, other groups of nitidulids have also independently established specialized relationships with male inflorescences of palms. For example, members of the Epuraeinae, such as all representatives of the Afrotropical subgenus *Apria* Grouvelle, 1919 (within the large and heterogeneous genus *Epuraea* Erichson, 1843, strongly needing a complete revision), are known to frequent palm inflorescences [62,107]. Similarly, other Epuraeinae of the same genus have been reported as regular pollinators of the Oriental palm *Nypa fruticans* Wurmb. [108,109,110]. A particularly isolated lineage, the subfamily Cillaeinae Kirejtshuk & Audisio, 1986, includes some tropical members typically associated as both larvae and adults with senescent palm stalks and inflorescence sheaths, where they primarily feed on different genera and species of filamentous fungi, being rarely found on fresh palm inflorescences [17,89,111,112]. Interestingly, recent studies on the insect pollinators of the Indonesian and Philippine palm *Nypa fruticans* Wurmb. report, in addition to *Epuraea* spp., some other unidentified Nitidulidae, including members of the above cited subfamily Cillaeinae (likely belonging to the genus *Brachypeplus* Erichson, 1842) [108,109,110].

## 7. Conclusions

Table 1 summarizes the most updated information on the ecological (demonstrated or inferred) relationships between Meligethinae and palms, and it includes several unpublished data on both described and undescribed pollen beetle species. As discussed throughout this review, the probably monophyletic meligethine group first associated with Arecaceae likely originated from a sudden “host jump” from dicots to monocots approximately 20 Mya. This event subsequently led to the emergence and diversification of the entire clade of the Palearctic *Meligethes*-complex of genera, later followed by a likely retrograde host shift back to dicotyledons. Notably, this latter shift involved plant families (Rosaceae, Brassicaceae, and Cleomaceae) that had not previously, or elsewhere, been colonized by the Meligethinae. In the meantime, the aforementioned “host jump” may have enabled the Meligethinae to exploit a newly available, ecologically open plant lineage—the Arecaceae—which was largely free of both natural enemies and competitors, thereby triggering a rapid adaptive radiation of this small pollen beetle lineage. As a result, nearly forty species and around ten genera are now known to be associated with a wide variety of phylogenetically diverse and pre-existing palm taxa. This hypothesis is consistent with recent studies showing that coevolution between hosts and their parasites is rarely a major driver of speciation [37,106,113,114,115]. Notable exceptions can be represented by cases of obligate pollination mutualism with highly specialized partners [92,93,94,116]. In line with this view, we have highlighted how certain palm species (e.g., *Elaeis guineensis* and *Phoenix reclinata* in tropical Africa: Table 1) [18,62] can host both larvae and adults of up to five species of *Meligethinus*, even on the same individual palm (Figure 6b). These co-occurring beetle species are often not closely related, further suggesting a lack of strict host-specific coevolution [55,117]. These observations support a model in which the evolution of Meligethinae on palms has likely been shaped by a combination of independent allopatric speciation events, secondary sympatry through range expansion, and repeated host shifts among (related and unrelated) already well-differentiated palm species. Similar evolutionary scenarios were recently observed in other unrelated pollinator beetle lineages, such as several members of the weevil tribe Derelomini [106,113,114,115]. Members of this lineage have been listed as a typical example of “brood-site pollination mutualism”—or nursery pollination, BSPM—a concept recently discussed by Haran et al. [106] to indicate an insect–host plant system where immature stages of a pollinator develop within tissues (either flowers, ovules, or pollen) of a plant as a reward for its pollination (made by the flying adult individuals of the involved species); this same concept can easily be applied to all palm-associated Meligethinae.

We also emphasize that the actual diversity of the Meligethinae associated with palms is likely severely underestimated. This underestimation is primarily due to a lack of targeted field research, the rarity of many palm species, and their often brief and unpredictable flowering periods, which hinder regular insect sampling.

Finally, we highlight the ecological and economic importance of certain meligethine species as pollinators—not only for agricultural and ornamental palms but also for species of high conservation concern. Further studies are clearly needed to assess their pollination efficiency, the degree of specialization (monophagy vs. oligophagy) [118,119,120,121], and their ecological uniqueness in palm pollination systems [122].

## Figures and Tables

**Table 1:** Genera and species of known or inferred palm-associated Meligethinae, with relevant information on their geographical distribution, habitat, altitude, phenology, and larval host plants. Genera are listed in a tentative phylogenetic order, while species within each genus are arranged alphabetically. Phenological data refers primarily to specimens collected on the inflorescences of host plants in order to reduce the influence of incidental findings outside their actual reproductive period. Several genera are here retained in their original generic rank, disregarding the unjustified and overly simplified synonymies with the genera *Microporum* C. Waterhouse, 1876 (*Lechanteuria*), *Cornutopria* S. Endrödy-Younga, 1978 (*Palmopria*), or *Cryptarchopria* Jelínek, 1975 (*Horakia*, *Kabakovia*), as proposed by Kirejtshuk and Kirejtshuk [40]. Bionomical, phenological, and distributional data is derived from literature sources [12,13,15,16,17,18,40,50,51,52,53,59,60,62,63,64,65] and is integrated with unpublished data [Audisio et al., unpublished].

Genera and Species	Distribution *habitat* (Phenology) Altitude	Larval Host Plant(s) (Arecaceae)
***Microporodes*** S. Endrödy-Younga, 1978	**Madagascar** ***tropical forests***	**Arecaceae**
*Microporodes dispar* (Murray, 1864)	Madagascar (**VII–VIII**) (300–600 m)	*Elaeis guineensis* Jacq.
***Palmopria*** S. Endrödy-Younga, 1978	**Tropical Africa** ***tropical forests***	*Elaeis guineensis* Jacq.
*Palmopria congolensis* (Grouvelle, 1915)	Tropical western Africa (from Sierra Leone and Togo to Democratic Republic of Congo and Angola) (**X–V**) (0–1000 m)	*Elaeis guineensis* Jacq.
*Palmopria elaeidis* S. Endrödy-Younga, 1978	Tropical western Africa (at least from Togo to Democratic Republic of Congo and Angola) (**X–II**) (0–1600 m)	*Elaeis guineensis* Jacq.
*Palmopria tomentosa* S. Endrödy-Younga, 1978	Tropical western Africa (at least from São Tomé to Democratic Republic of Congo and Angola) (**X–II**) (0–1200 m)	*Elaeis guineensis* Jacq.
***Cornutopria*** S. Endrödy-Younga, 1978	**Democratic Republic of Congo** ***tropical forests***	**Probably Arecaceae but formally unknown**
*Cornutopria basilewskyi* S. Endrödy-Younga, 1978	As above (**VIII–IX**) (300–500 m)	unknown
***Lechanteuria*** S. Endrödy-Younga, 1978 **^(1)^**	**Tropical western Africa** (Guinea to Democratic Republic of Congo) ***tropical forests***	**Probably Arecaceae or Moraceae but unknown with certainty ^(2)^**
*Lechanteuria binotata* (Lechanteur, 1955)	Democratic Republic of Congo (**VIII–IX**) (200–500 m)	Unknown **^(2)^**
*Lechanteuria corbisieri* (Kirejtshuk, 1980) **^(1)^**	Democratic Republic of Congo (**VIII–IX**) (200–500 m)	Unknown **^(2)^**
*Lechanteuria interrupta* (Kirejtshuk, 1980) **^(1)^**	Democratic Republic of Congo (**IX–X**) (800–1000 m)	Unknown
*Lechanteuria* sp. (Audisio et al., unpublished) ^(**3)**^	Guinea (**IX**) (1400 m)	Unknown
***Cryptarchopria*** Jelínek, 1975	**Oriental Region** ***tropical forests***	**Various genera and species of Arecaceae**
*Cryptarchopria infima* (Grouvelle, 1895)	Indonesia (Java, Moluccas Islands) (**X–XI**) (0–500 m)	*Areca catechu* L.
*Cryptarchopria kabakovi* Kirejtshuk, 1979	Vietnam (**III–VI**) (0–200 m)	*Arenga* spp.
*Cryptarchopria ponomarenkoi* Kirejtshuk, 1989	Vietnam, N Thailand (**V–VI**) (1000–1500 m)	*Caryota mitis* Lour.
*Cryptarchopria* sp. nov. 1 (Jelínek, unpublished) **^(4)^**	Indonesia, Sangir Island (=Sangihe Island) (**XI**) (200–600 m)	Almost certainly Arecaceae but formally unknown
***Horakia*** Jelínek, 2000	**NW Thailand, and border areas between SW China and the** **E Arunachal-Pradesh (NE India)** ***subtropical mountain forests***	**Arecaceae (maybe all on *Caryota* spp.)**
*Horakia kubani* Jelínek, 2000	NW Thailand (**V–VI**) (1100–1600 m)	Probably *Caryota obtusa* Griff. (=*C. gigas* Hahn ex Hodel)
*Horakia* sp. nov. 1 (Liu et al., unpublished) **^(5)^**	Southern-western China (Tibet, Medog County) (**VII**) (1400–1500 m)	*Caryota maxima* Blume
*Horakia* sp. nov. 2 (Lasoń et al., unpublished) **^(5)^**	Border area between SW China and the E Arunachal-Pradesh (NE India) (**V–VI**) (1500–1800 m)	Unknown, maybe *Caryota* sp.
***Kabakovia*** Kirejtshuk, 1979	**Oriental and Afrotropical Regions** ***tropical and subtropical forests***	***Phoenix* spp. and other Arecaceae**
*Kabakovia ivoriensis* (Kirejtshuk & Kirejtshuk, 2012) **^(6)^**	Ivory Coast (**XI–XII**) (0–200 m)	Probably *Borassus akeassii* Bayton, Ouédr. & Guinko
*Kabakovia latipes* (Grouvelle, 1908)	India, Sri Lanka, Nepal, Vietnam (**III–VI**) (0–1800 m)	*Phoenix loureiroi* Kunth (=*P. humilis* and *P. hanceana*)
*Kabakovia nepalensis* (Kirejtshuk & Kirejtshuk, 2012)	Nepal (**VIII–IX**) (150–300 m)	Unknown but probably *Borassus flabellifer* L.
*Kabakovia* sp. **^(6)^**	Uganda (**IV**) (1200 m)	Unknown but probably *Borassus aethiopum* Mart.
***Meligethinus*** Grouvelle, 1906	**Oriental, Afrotropical, and southern Palearctic Regions** ***tropical forests, suberemic areas, Mediterranean shrublands***	**Several unrelated genera of Arecaceae**
*Meligethinus apicalis* (Grouvelle, 1894)	N India (W Bengal), SW China ***tropical forests*** **(unknown)**	Unknown
*Meligethinus bisignatus* Kirejtshuk, 1980	Democratic Republic of Congo, Rwanda ***tropical forests and shrublands*** (**I–II, VII–VIII**) (900–1500 m)	*Elaeis guineensis* Jacq.
*Meligethinus dolosus* Grouvelle, 1919	NE South Africa, S Mozambique ***tropical forests and shrublands*** (**VIII–X**) (0–500 m)	*Phoenix reclinata* Jacq.
*Meligethinus gedrosiacus* Jelínek, 1981	Iran, E Arabian Peninsula ***suberemic areas*** (**IV–V**) (0–1300 m)	*Nannorrhops ritchiana* (Griffith) Aitch
*Meligethinus grouvellei* Kirejtshuk, 1980 **^(7)^**	Southern and eastern India ***tropical forests*** (**unknown**)	Unknown
*Meligethinus hamerlae* Sabatelli et al., 2020	S Mozambique (Inhaca Island) ***tropical forests and shrublands*** (**VIII–X**) (0–20 m)	*Phoenix reclinata* Jacq.
*Meligethinus humeralis* Grouvelle, 1906	Angola, Democratic Republic of Congo, Rwanda, Mozambique ***tropical forests and shrublands*** (**I–II, VII–IX**) (0–1300 m)	*Phoenix reclinata* Jacq.
*Meligethinus kabakovi* Kirejtshuk, 1980	Vietnam, S China including Taiwan ***tropical forests*** (**II–III**) (0–200 m)	Probably *Chuniophoenix* spp.
*Meligethinus mondlanei* Sabatelli et al., 2020	S Mozambique ***tropical shrublands*** (**VIII–X**) (0–20 m)	*Phoenix reclinata* Jacq.
*Meligethinus muehlei* Jelínek, 1992	Rwanda ***tropical forests*** (**I–II**) (0–1500 m)	*Elaeis guineensis* Jacq.
*Meligethinus pallidulus* (Erichson, 1843)	W Mediterranean areas ***Mediterranean maquis*** (**III–VI**) (0–2200 m)	*Chamaerops humilis* L
*Meligethinus peringueyi* (Grouvelle, 1919)	NE South Africa, S Mozambique ***tropical shrublands*** (**VIII–X**) (0–500 m)	*Phoenix reclinata* Jacq.
*Meligethinus plagiatus* (Grouvelle, 1894)	N India (W Bengal), Vietnam, S China including Taiwan ***tropical forests*** (**IV–VI**) (0–500 m)	Probably *Chuniophoenix* spp.
*Meligethinus quadricollis* Kirejtshuk, 1987	N India (Uttarakhand) ***tropical forests*** (**unknown**)	Unknown
*Meligethinus singularis* (Grouvelle, 1919)	NE South Africa ***tropical shrublands*** (**unknown**)	Probably *Phoenix reclinata* Jacq. or *Hyphaene petersiana* Klotzsch ex Mart.
*Meligethinus* sp. 1 (Audisio et al., unpublished) **^(8)^**	South Africa (Eastern Cape) ***tropical forests*** (**XI**) (0–200 m)	Probably *Jubaeopsis caffra* Becc. or *Hyphaene petersiana* Klotzsch ex Mart.
*Meligethinus* sp. 2 (Audisio et al., unpublished) **^(8)^**	E Madagascar ***tropical forests*** (**II**) (1000 m)	Unknown, probably *Dypsis* sp. or *Ravenea* sp.
*Meligethinus suffusus* Kirejtshuk, 1980	Democratic Republic of Congo, Mozambique, NE South Africa ***tropical forests*** (**I–V**, **VIII–X)** (0–2000 m)	*Phoenix reclinata* Jacq. and likely other forest Arecaceae
*Meligethinus tschungseni* Kirejtshuk, 1987	S and Central China, N Vietnam, NE India, Japan ***subtropical forests*** **(IV–VII)** (100–2000 m)	*Trachycarpus fortunei* (Hook.) H. Wendl.
*Meligethinus zimbabwensis* Kirejtshuk, 2011	W Zimbabwe ***subtropical forests*** **(XII)** (600–800 m)	Probably *Phoenix reclinata* Jacq. or *Hyphaene petersiana* Klotzsch ex Mart

**^(1)^** The recent re-examination by co-author PA of the type material of *Prianella binotata* Lechanteur, 1955 [*Lechanteuria binotata* (Lechanteur, 1955)], *Microporum corbisieri* Kirejtshuk, 1980, and *Microporum interruptum* Kirejtshuk, 1980 (deposited in the Royal Museum for Central Africa, Belgium—MRAC) confirmed the clear generic distinction of the African genus *Lechanteuria* S. Endrödy-Younga, 1978. The previously proposed synonymy with *Microporum* C. Waterhouse, 1876 (whose species are restricted to Madagascar, the Comoros Islands, and Aldabra, and are associated with Pandanaceae) was incorrectly introduced by Kirejtshuk and coauthors [40,53,66]. Furthermore, the two above listed species of *Microporum* described by Kirejtshuk [53] from the former Zaire should also be reassigned to *Lechanteuria*. These taxonomic revisions will be formally addressed in a forthcoming article on the higher systematics of the Meligethinae (Audisio et al., in prep.). **^(2)^** Based on the same original source [MRAC], various authors [15,53,64] reported that some specimens of *Lechanteuria* were collected on fruits of *Treculia engleriana* (now *Treculia africana* Decne. ex Trécul; Moraceae). Given that no Meligethinae are known be carpophagous in any way, at least during the larval stage, two scenarios may be proposed: (1) the presence of *Lechanteuria* adults on *Treculia* fruits was incidental, possibly related to the intake of sugary exudates in the absence of flowering structures from their true host plants, likely Arecaceae; (2) the collectors may have misidentified the globular *Treculia* inflorescences as fruits. In this latter case, it cannot be excluded that Moraceae may indeed serve as larval host plants for *Lechanteuria* species; if so, members of this genus, morphologically closely related to others strictly associated with palms, may actually have experienced a further “host jump” to Moraceae. **^(3)^** This small-sized mountain species, recently discovered in Guinea, will be described in a forthcoming publication by Audisio et al. (unpublished data). **^(4)^** This newly discovered species from Indonesia will be described in a forthcoming publication by Jelínek et al. (unpublished data). **^(5)^** These two highly distinctive undescribed species—the second one notable for the exceptional development of the head and antennae in males—were recently discovered in the border region between southwestern China and eastern Arunachal Pradesh, northeastern India. They will be described in a forthcoming publication by Liu et al., following an upcoming research mission to southwestern China aimed at identifying the host plant of the second species, and collecting fresh material for molecular analyses. **^(6)^** Some distinctive morphological traits observed in *Kabakovia nepalensis* and in the African species of *Kabakovia*—one described from Ivory Coast as *K. ivoriensis* by Kirejtshuk and Kirejtshuk [40]—and another one, closely related to the latter, recently discovered in museum material from Uganda (to be treated in a forthcoming publication by Audisio et al.) (Figure 4), suggest that these taxa may represent a lineage closely related to, but perhaps distinct from, the true Indochinese *Kabakovia* (*K. latipes*). **^(7)^** The taxonomic position of this Oriental taxon will be thoroughly discussed in an upcoming revision of *Meligethinus* (Liu et al., in prep.). **^(8)^** These new species from eastern South Africa (Eastern Cape) and Eastern Madagascar will be described in the aforementioned forthcoming revision of the genus *Meligethinus* (Liu et al., in prep.).
